# Supplementary figures and images for: Globoside and the mucosal pH mediate parvovirus B19 entry through the epithelial barrier
Source: PLoS Pathog. 2023 May 23;19(5):e1011402. doi: 10.1371/journal.ppat.1011402 (PMC10241365; doi:10.1371/journal.ppat.1011402)

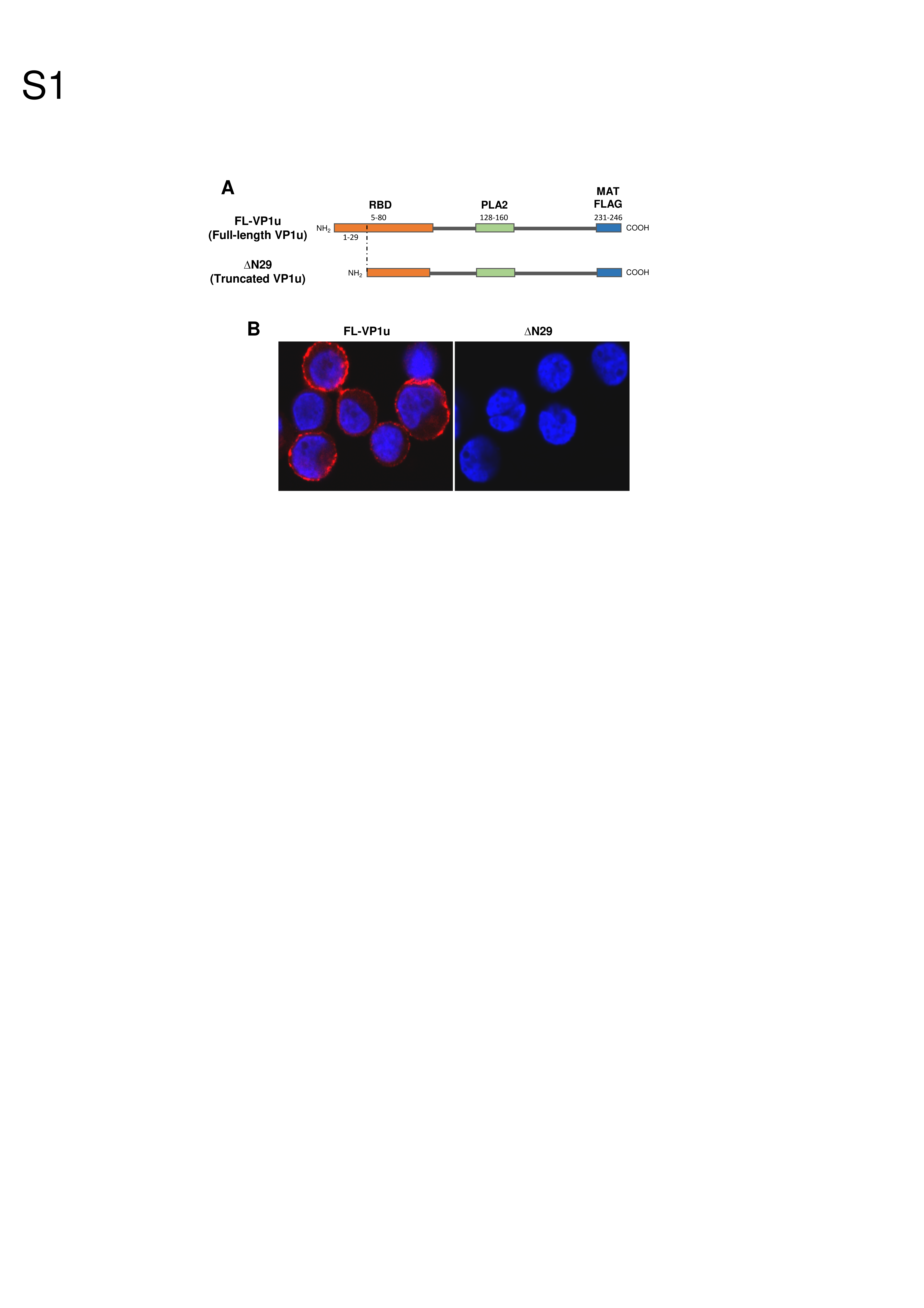

Supplement: S1 Fig — (A) Schematic representation of functional (FL-VP1u, Full-length) and non-functional (∆N29, N-terminal truncated) recombinant VP1u constructs. RBD, receptor-binding domain. PLA2, phospholipase A2. MAT, metal affinity tag (B) Detection of the VP1u receptor in UT7/Epo cells. Cells were incubated with recombinant VP1u constructs and an anti-FLAG antibody, washed, fixed, and visualized by confocal microscopy. DAPI was used to visualize nuclei. (TIF) [file ppat.1011402.s001.tif]

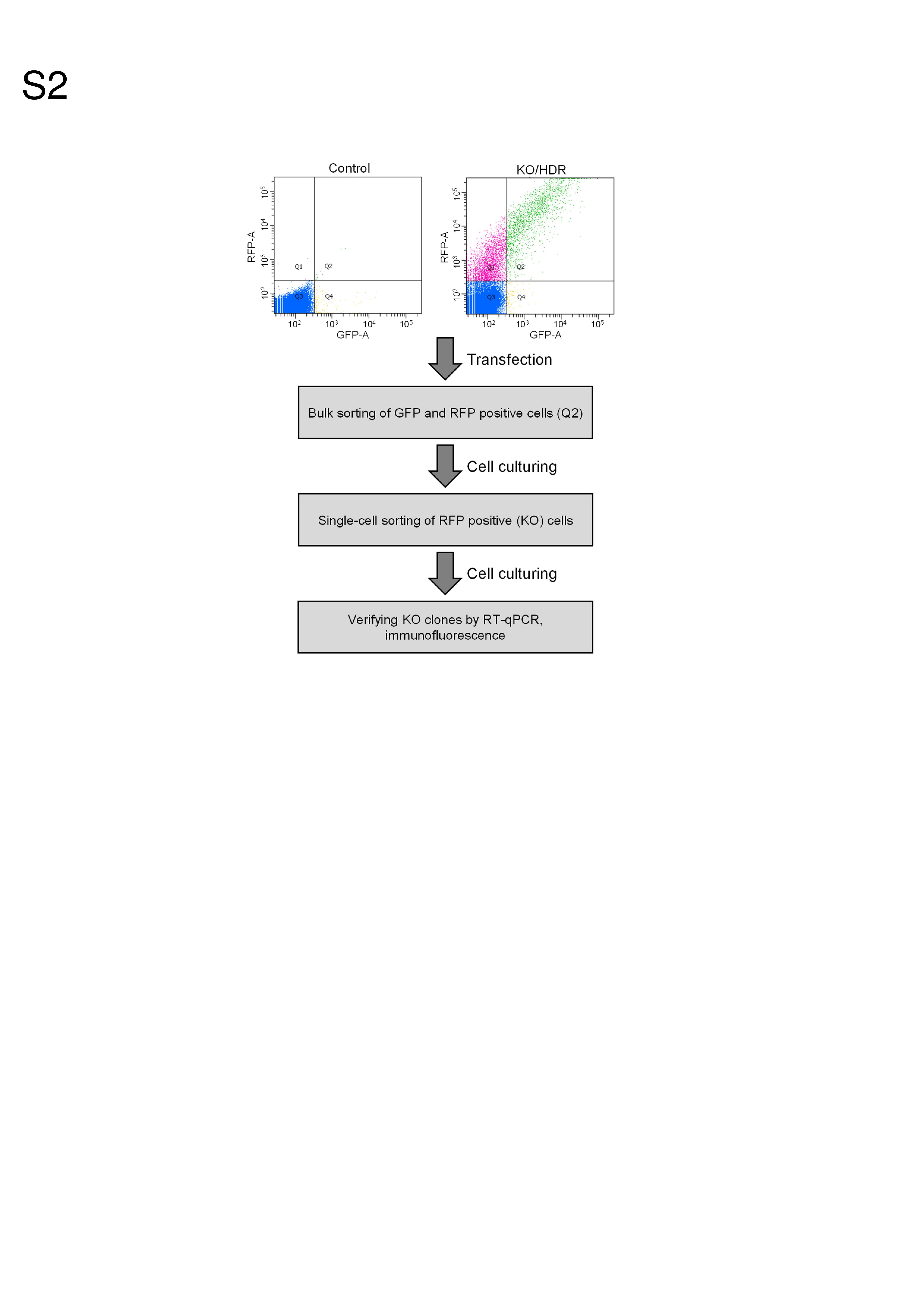

Supplement: S2 Fig — MDCK II cells were transfected with either control or B3GalNT1 KO and HDR plasmids and analysed by FACS. Cells containing both CRISPR/Cas9 KO and HDR plasmids (Q2) showed green fluorescence protein (GFP), and red fluorescence protein (RFP) expression, respectively. These cells were sorted and cultured for one month. Single-cell sorting was performed to obtain clones with stably integrated HDR cassettes expressing RFP. Successful knockout of B3GalNT1 was verified by RT-qPCR and immunofluorescence staining with an antibody against globoside. (TIF) [file ppat.1011402.s002.tif]

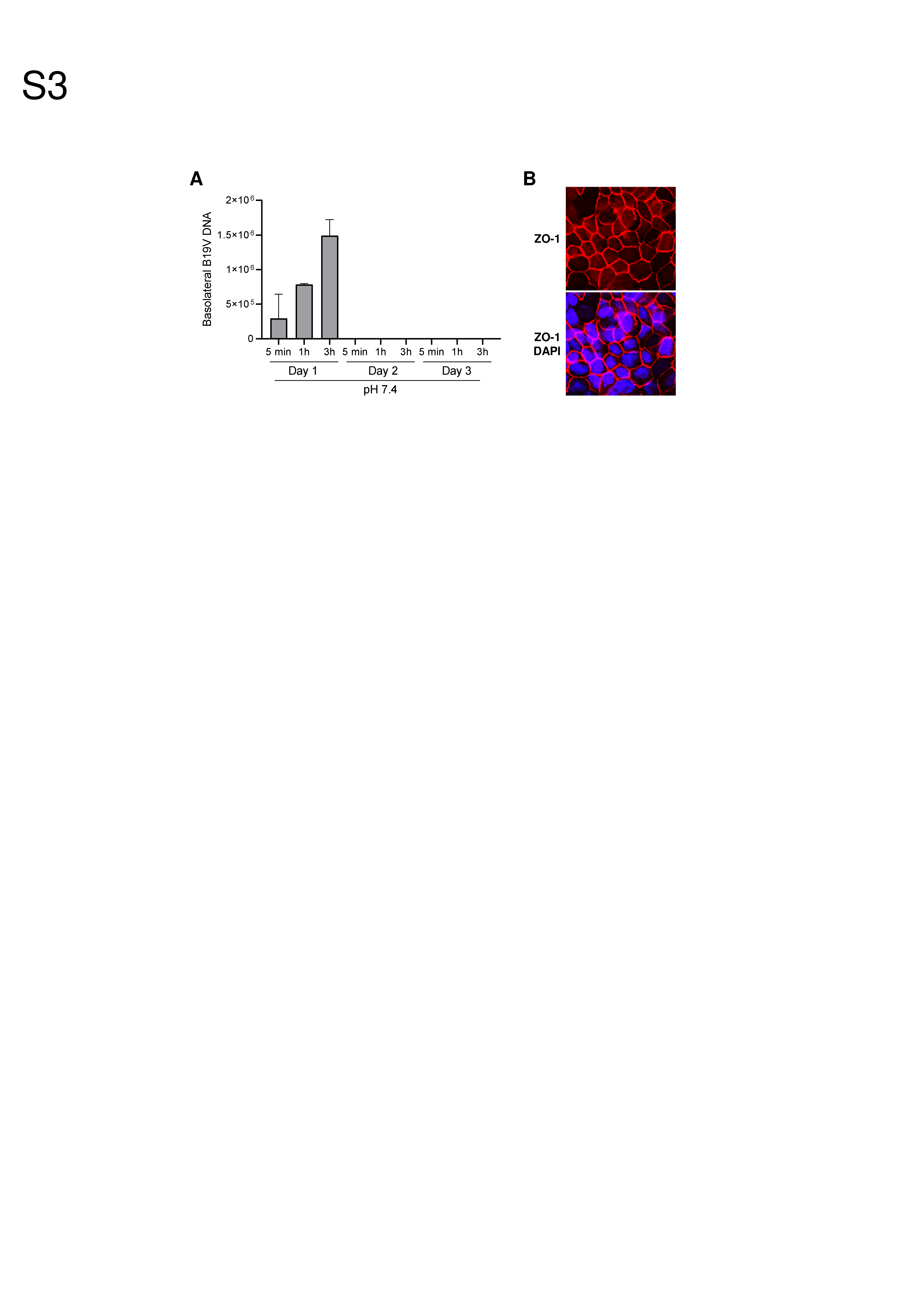

Supplement: S3 Fig — (A) MDCK II cells were seeded in TC inserts and allowed to form a polarized monolayer. To evaluate paracellular transfer, B19V (3x10^9^) was added to the apical medium at neutral pH at progressive days post-seeding. At the indicated hours post-infection, viruses were quantified in the basolateral medium by qPCR. (B) At 3 days post-seeding, the formation of tight junctions was visualized with an antibody against ZO-1 and a secondary Alexa Fluor 594. DAPI was used to visualize nuclei. (TIF) [file ppat.1011402.s003.tif]

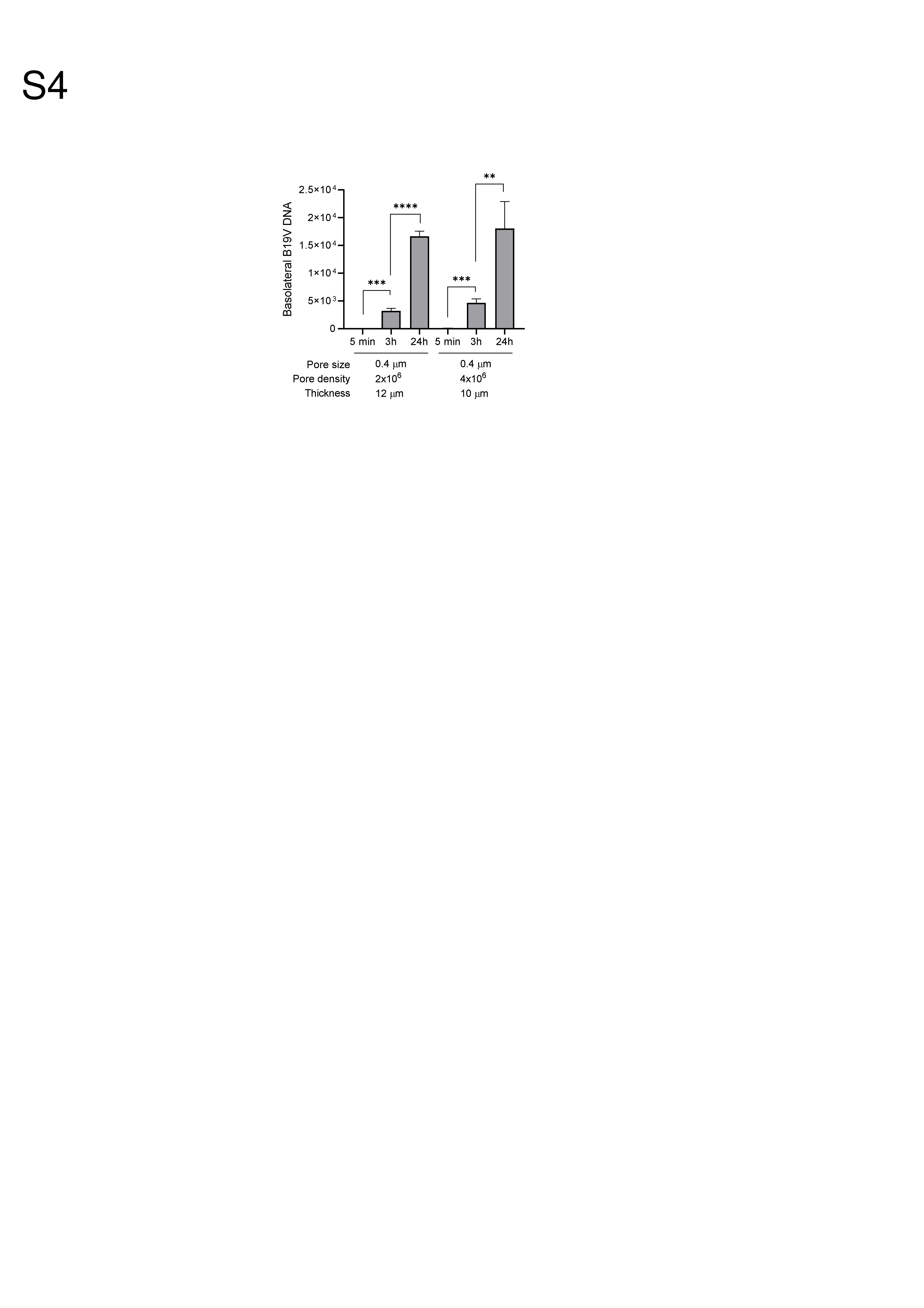

Supplement: S4 Fig — Membranes with varaying pore densities and thicknesses were tested in parallel. B19V was added to the apical side of the MDCK II cells at acidic pH (6.1), and the accumulation of viruses in the basolateral medium was quantified at increasing times post-inoculation by qPCR. (TIF) [file ppat.1011402.s004.tif]

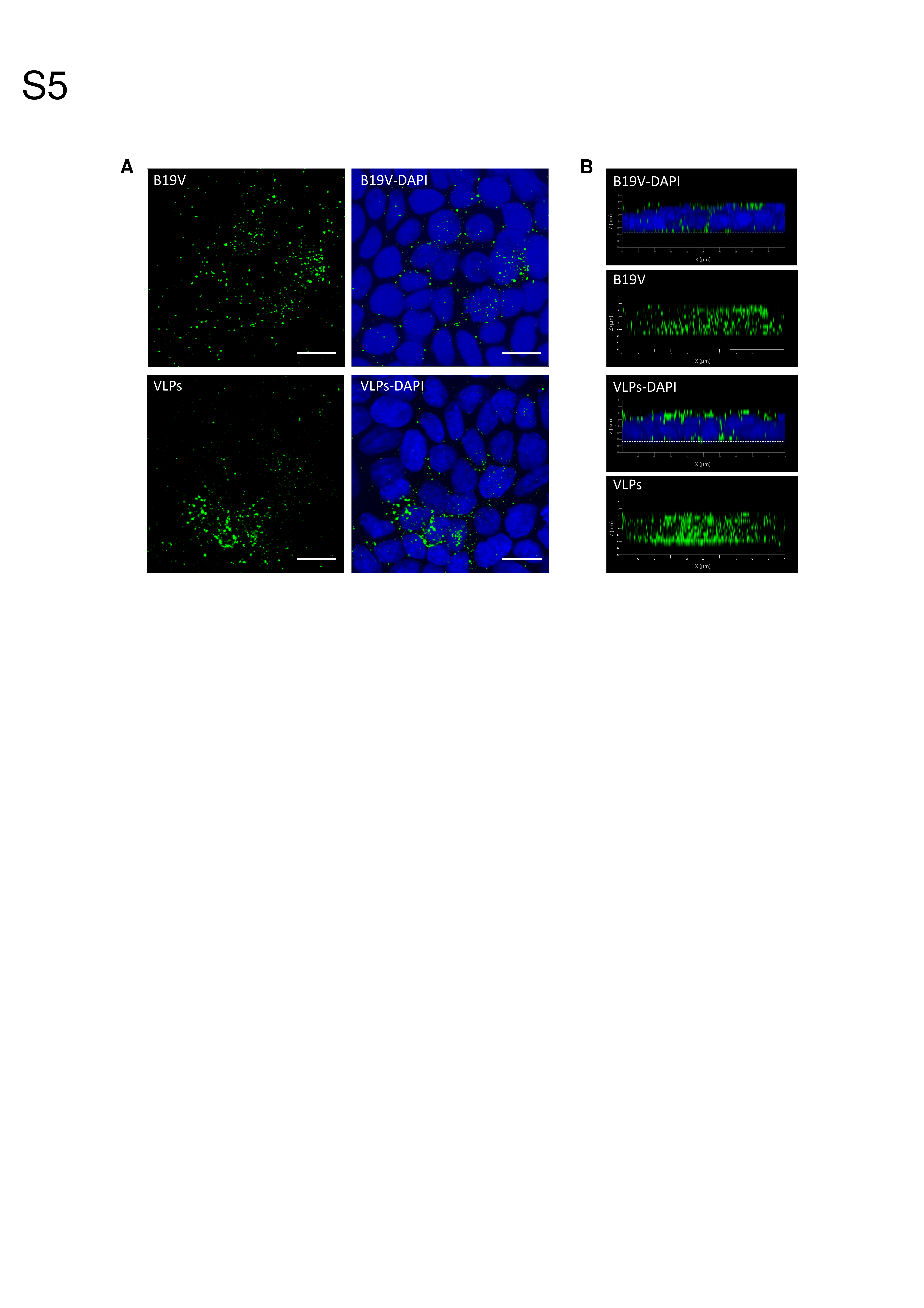

Supplement: S5 Fig — Polarized MDCK II cells were grown on TC inserts for 3 days to allow the formation of tight junctions. B19V or VLPs (3x10^9^) were added at 37°C for 1h at pH 6.1. The cells were washed, fixed, and stained with an antibody against intact capsids (green). Nuclei were stained with DAPI (blue). Z-stacks were acquired by confocal microscopy from the top of the epithelial layer to the beginning of the porous membrane (dotted line). (A) Z-projections (XY). (B) Orthogonal view (XZ). Scale bar: 10 μm. (TIF) [file ppat.1011402.s005.tif]

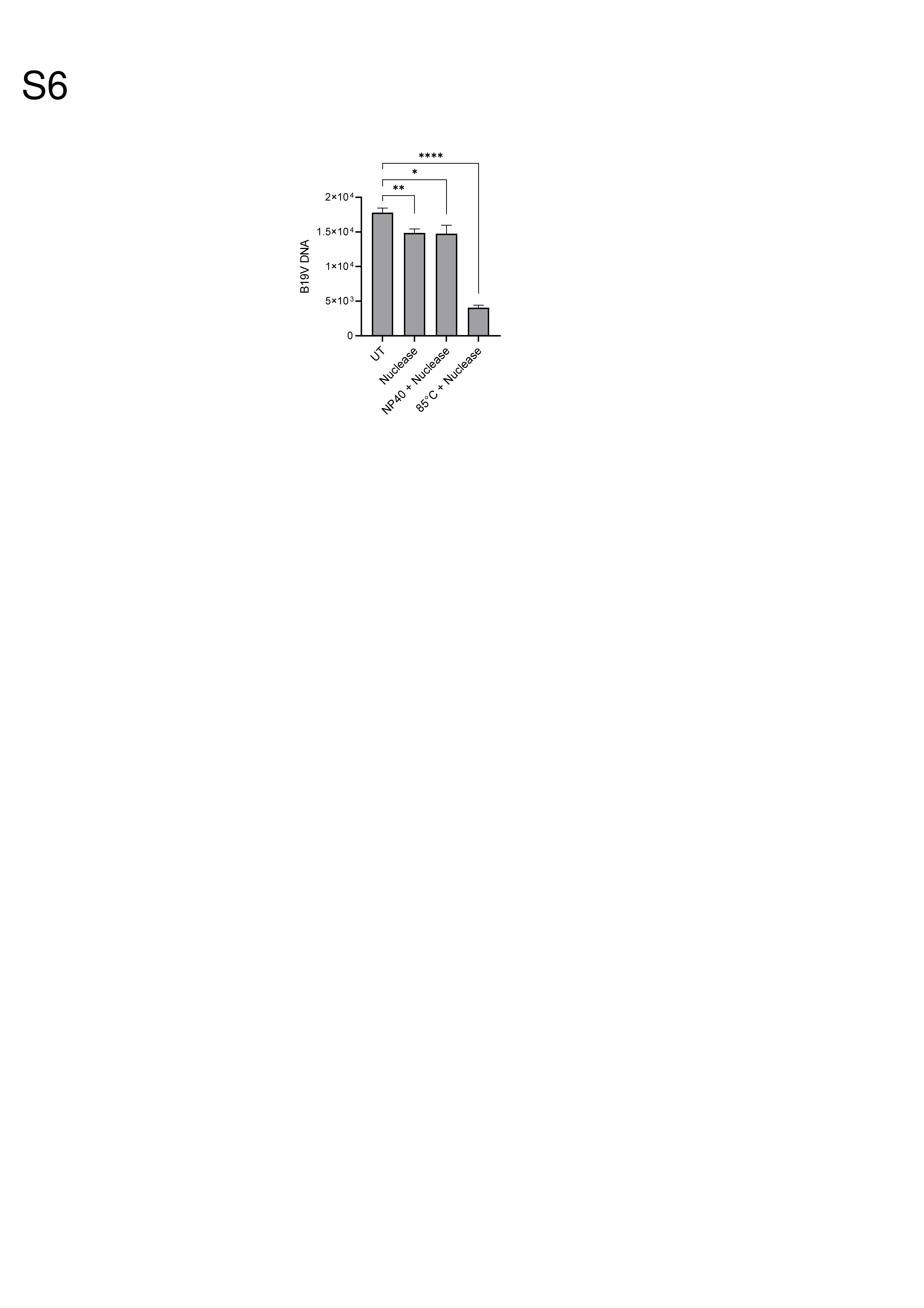

Supplement: S6 Fig — Following virus transcytosis, the basolateral medium was treated with micrococcal nuclease alone or in combination with NP40 prior to DNA extraction and qPCR. Basolateral media (100 μl) was incubated with 11 μl of 10X nuclease buffer (500 mM Tris-HCl pH 8.0, 150 mM CaCl2), 1.1 μl of 10 mg/ml BSA, and 0.2 μl micrococcal nuclease (400 units, NEB) at 37°C for 1h. The reaction was quenched with 11 μl 0.2 M EDTA. Alternatively, nuclease treatment was performed in combination with 0.1% NP40. As a control, the samples were heated to 85°C for 5 minutes prior to nuclease digestion to expose the viral DNA. UT, untreated. (TIF) [file ppat.1011402.s006.tif]

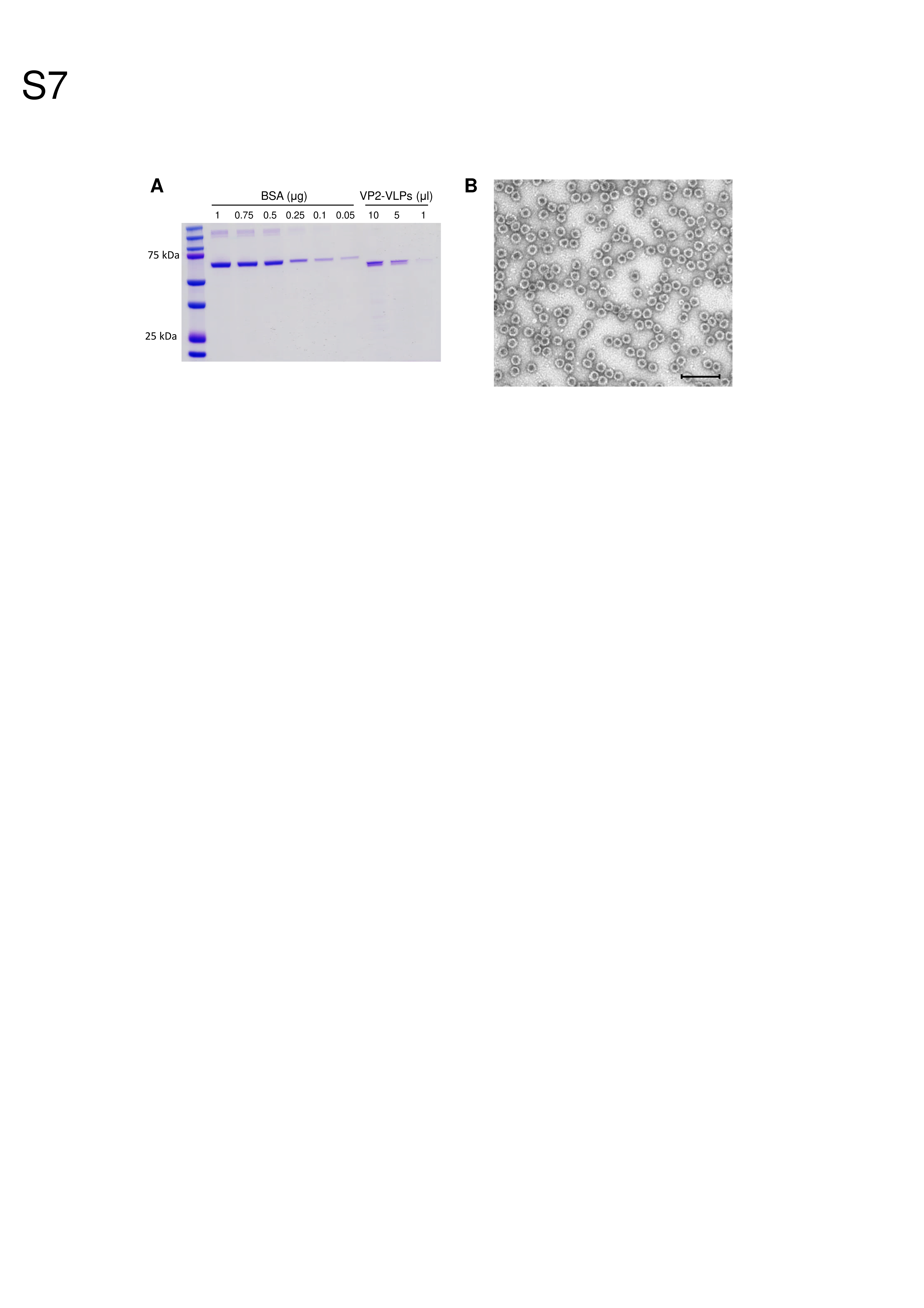

Supplement: S7 Fig — (A) The purity and concentration of VLPs (VP2-only particles) were examined by SDS-PAGE. Protein concentration was determined using serial dilutions of purified BSA. (B) The integrity and monodispersity of VLPs were analyzed by electron microscopy. Scale bar; 100 μm. (TIF) [file ppat.1011402.s007.tif]

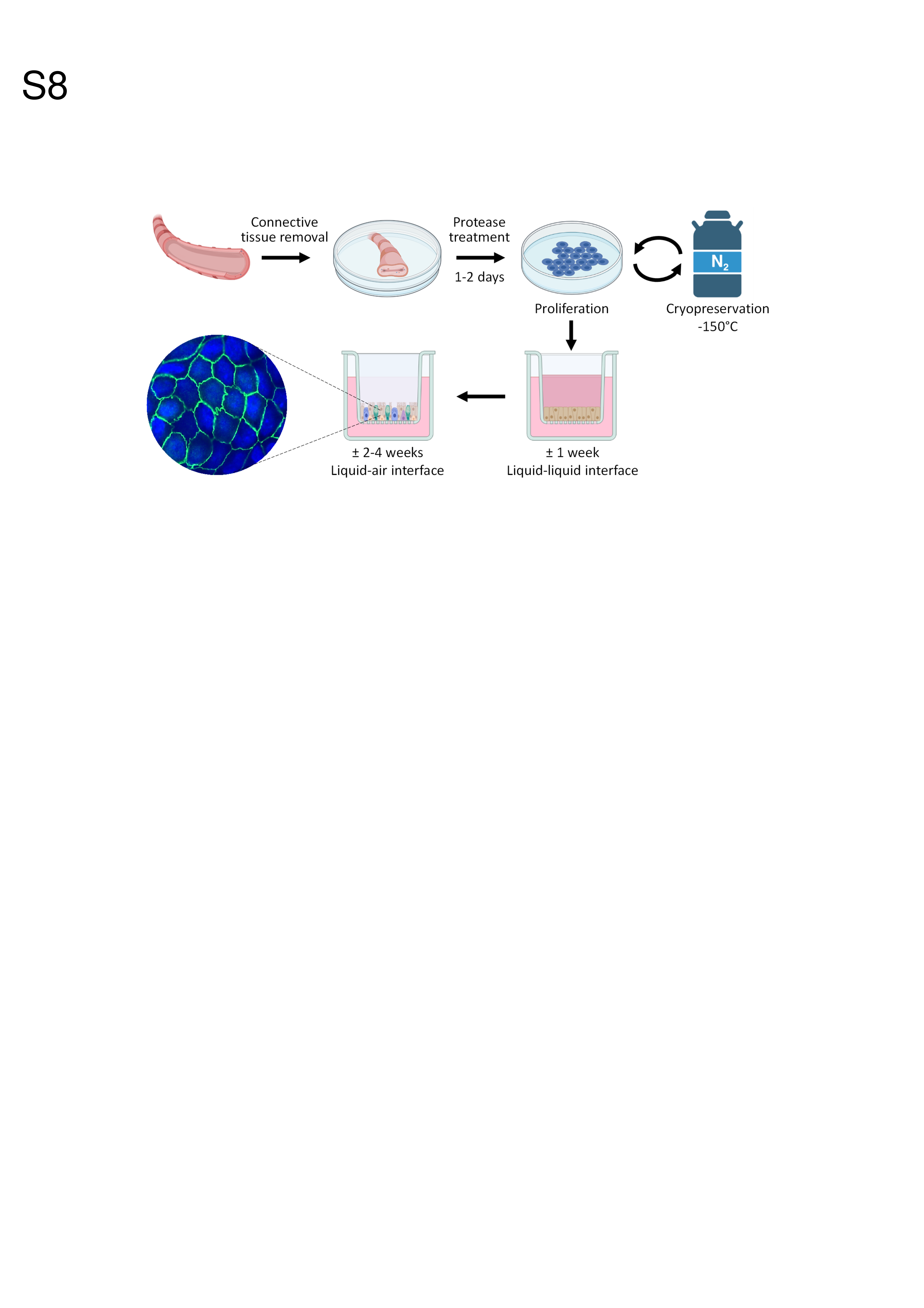

Supplement: S8 Fig — Primary epithelial cells are isolated from tissue biopsies, from which connective tissue is removed, using protease treatment, and grown in BEGM media to expand the cells as a monolayer. Expanded cells are seeded onto Transwell membranes and grown under the submerged condition until they reach confluence. The differentiation phase is initiated by air-lifting the cells to establish the air-liquid interface (ALI). During the 4 weeks post-ALI exposure, the AEC cultures will differentiate into a pseudostratified layer of differentiated AEC cultures, showing the phenotype of ciliated, goblet, and basal cells. Created with BioRender.com. (TIF) [file ppat.1011402.s008.tif]

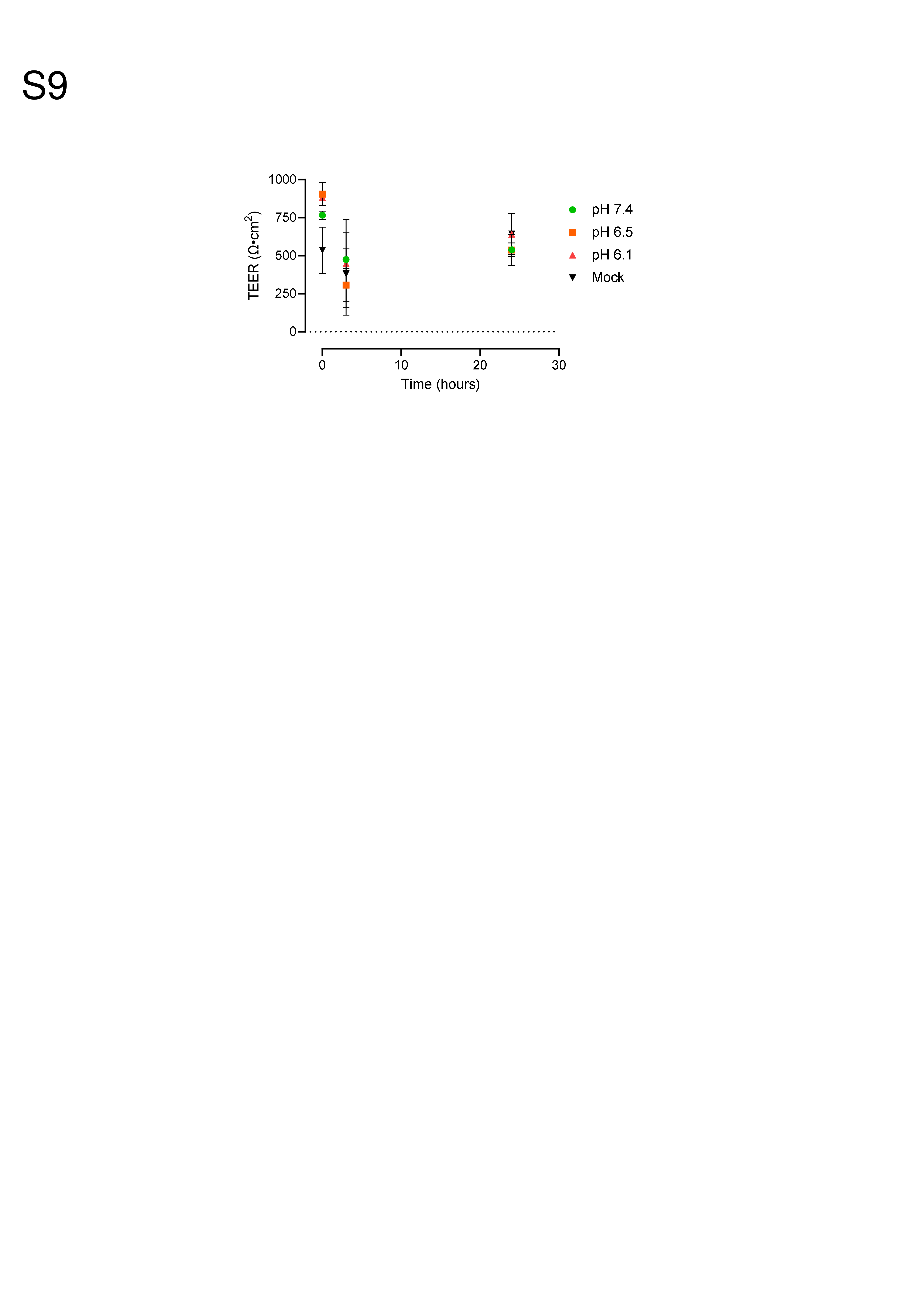

Supplement: S9 Fig — The apical side of hAECs was exposed to different pH conditions and infected with B19V at 37°C. At increasing post-infection times, TEER was measured manually using an Epithelial Volt/Ohm Meter (EVOM) with a “chopstick” electrode (World Precision Instruments, Hitchin, UK). A volume of 200 μl of TEER solution (4.5 g NaCl, 91.89 mg CaCl2, and 1.194 g of HEPES in 500 ml of distilled water) was added to the apical chamber of the Transwells before inserting the electrodes. (TIF) [file ppat.1011402.s009.tif]
