# Supplementary material for: Globoside and the mucosal pH mediate parvovirus B19 entry through the epithelial barrier
Source: PLoS Pathog. 2023 May 23;19(5):e1011402. doi: 10.1371/journal.ppat.1011402 (PMC10241365; doi:10.1371/journal.ppat.1011402)
Supplement: Attachment — Submitted filename: Response to Reviewers.pdf [file ppat.1011402.s010.pdf]

Reviewer's Responses to Questions

Part I - Summary
Please use this section to discuss strengths/weaknesses of study, novelty/significance, general execution and scholarship.

Reviewer #1: Corinne Suter and her colleagues have elucidated the mechanism by which human parvovirus B19 is transcytosed via globoside in epithelial cells. Initially, they demonstrated that binding of B19V particles to the cell surface is essential for their internalization using MDCKII cells, and this process is VP1u-independent but dependent on low pH conditions. Additionally, they demonstrated that the binding process is reliant on both globoside and caveolae endocytosis. Moreover, they observed that this transcytosis mechanism was also present in primary human airway cells. The route of airborne transmission of B19V to target erythroid progenitor cells has not been well understood. The study has addressed a significant topic regarding the invasion of B19V virions via transcytosis of respiratory epithelial cells. Overall, the experiments were executed, and the outcomes were presented clearly. I have a few suggestions to make the message clearer and provide some useful additional information.

Reviewer #2: In their previous publications (Bieri et al. 2019, 2021) the authors demonstrated that globoside is dispensable for the B19V entry and that the acidic pH is required for the B19V attachment to globoside and this interaction is required for endocytic entry. This current paper is elucidating the role of an acidic environment for the transcytosis of B19V through epithelial model MDCK II cells and primary airway epithelial cells. These studies were performed with cells grown on porous membranes which allowed the detection of B19V entry from the apical and egress from the basolateral side. The absence of produced NS1 verified that the virus did not replicate in the cells during transcytosis. The characterization of the entry pathway suggested that B19V entry in acidic conditions is mediated by a caveolar-like entry pathway.

This is a compact, clearly written, and rather jargon-free paper. The performed experiments are well thought out, and they proceed logically answering the questions arising. However, there are major issues with the figures, as some of the figures do not correspond to the text, and figure legends, all in all complicating the revision of the reported results.

After modification, this publication is most likely highly useful for others wishing to study the regulation of viral particle transcytosis in polarized cells. Therefore, I believe this study makes a valuable contribution to the field of virus-cell interactions and how pathogenic viruses move across the barrier formed by epithelial cells using transcytosis.

Reviewer #3: B19V is a human pathogenic virus transmitted via respiratory route, although targeting erythroid progenitors in the bone marrow to achieve a productive infection. Portal and mode of entry have remained elusive so far, transcytosis being a likely mechanism. In their work, Sutter et al. present an abundant set of experimental data actually showing transcytosis of B19V through epithelial cells first in MDCK II as model, then in hAEC as a relevant system. Crucial to transcytosis, interaction of virions with globoside in acidic conditions, a key parameter for achieving a productive infection in the main target cells. Overall, the submitted paper provides convincing evidence and significantly contributes to our understanding of B19V interactions with target cells and mode of transmission, confirming the expertise of the group. Hereby, I provide some comments with the aim of a better readability and understanding of the paper.

Part II – Major Issues: Key Experiments Required for Acceptance
Please use this section to detail the key new experiments or modifications of existing experiments that should be absolutely required to validate study conclusions.

Generally, there should be no more than 3 such required experiments or major modifications for a "Major Revision" recommendation. If more than 3 experiments are necessary to validate the study conclusions, then you are encouraged to recommend "Reject".

Reviewer #1:

1- Line261-262: Performing a subcellular localization study through Z stack sectioning from apical to basolateral is crucial in highlighting the potential intracellular accumulation of B19V in MDCKII cells.

S5 Fig: Z-stack sectioning from apical to basal has been performed in MDCK II cells incubated with B19V and with VLPs (lacking the entire VP1), and it is presented as supplementary figure S5.

L196-197: As expected, infectious particles and VLPs are internalized and appear distributed across the cellular barrier, from the apical to the basal side.

2- In Fig7, it is essential to include the uptake of VLPs (VP2 only) via clathrin-independent, cholesterol, and dynamin-dependent mechanisms in primary human airway cells, which excludes VP1u involvement.

VP1u cannot be involved in the uptake process because it does not interact with hAECs.

This was confirmed by incubating hAECs cultures with functional recombinant VP1u constructs. As shown in Fig. 8B, VP1u does not interact with hAECs. In contrast, VP1u does interact with UT7/Epo (B19V target cells) cells and becomes internalized (S1 Fig).

To further clarify this, we have added the following sentence:

L343-345: "The VP1u erythroid receptor responsible for B19V internalization in EPCs was not detectable in the hAECs, excluding its role in virus uptake in the airway epithelium."

3- In Fig8, it is crucial to include the uptake and transcytosis of VLPs (VP2 only) under acidic conditions to determine if the VP2 interacts with globoside to mediate the novel uptake pathway in epithelial cells. Additionally, it is imperative to provide infectivity data of the B19V recovered in the basal medium following transcytosis through cells, as presented in the current fig3d.

We consider that these points have already been answered in this study and in our previous publication.

- In our previous publication (Bieri et al., PLoS Pathog. 2021;17: e1009434), we confirmed that globoside and acidic conditions mediate the binding and internalization of VLPs (VP2-only particles) into human erythroid progenitor cells, demonstrating that uptake is exclusively mediated by VP2 interaction with globoside. In this study, we have further confirmed the role of VP2, globoside and low pH in the interaction and uptake of VLPs in the canine epithelial cells MDCK II (Fig. 5A).
- The infectivity of the virus after transcytosis was already tested in MDCK II cells (Fig. 3D), and the integrity of the capsids following transcytosis was verified by exposure to detergents and nucleases (S4 Fig). Besides, detection of viral particles by IF during transcellular transport was always performed with a conformational-dependent mAb detecting exclusively intact capsids (mAb 860-55D; Gigler et al., J Virol. 1999;73:1974-9). This was mentioned throughout the manuscript.

4- Line 352-357: As mentioned by the authors, AXL was recently identified as a VP1u partner that facilitates B19V entry into cells. It is quite important to demonstrate that this event is distinct from AXL-mediated viral entry and to confirm that these cells do not express AXL on the cell surface.

Probably, the reviewer did not notice that the expression of VP1u receptor(s) in hAECs was tested with a recombinant VP1u (Fig. 8B). The result confirmed that hAECs do not express AXL, or any other VP1u-interacting molecule. Moreover, the lack of expression of AXL in hAECs was reported

elsewhere (Ruysseveldt et al., Front Allergy 2021: 787128), and the work was cited in the manuscript (L365-368).

Reviewer #2:

1. In general, the clathrin-independent, caveolin-like entry pathway of capsids from the apical side during transcytosis is well described and studied here. Does this rule out the autophagosome-associated pathway?

Autophagy is an intracellular process to degrade and recycle cellular components. Viruses can manipulate autophagy to promote replication. We cannot exclude that B19V manipulates autophagy in the epithelial cells to promote transcellular transport. However, autophagy is an intracellular process and does not directly concern uptake by clathrin or caveolae-mediated endocytosis, which is the specific step that we address in our study.

2. There is one aspect ongoing throughout the paper that is of concern, and that is the question of how does capsid exocytosis/exit from the basolateral side happen? Why does the acidic PH outside the cells affect the exit of the virus from the basolateral side? This important aspect is not addressed and should be introduced and discussed, and even experimented on.

The release of B19V into the basolateral medium is blocked when the basolateral pH is acid because, under acidic pH, the dissociation of the virus from globoside does not occur.

In previous studies, we found that pH acts as an affinity switch to regulate binding and dissociation between B19V and globoside. B19V-globoside complex dissociates when exposed to neutral pH (Bieri et al., PLoS Pathog. 2021;17: e1009434). This study was cited in our manuscript (L147). In this study, we confirmed our previous observations and found that exposure to neutral pH triggers the dissociation of the virus from globoside in epithelial cells (Fig. 1D and 1F). Dissociation does not occur at acidic pH.

3. Where is the virus blocked in absence of low pH? Specifically, lines 254-264, Figure 6B: What is the localization of the intact capsids - intracellular or on the cell surface? It seems that they might be located in intracellular vesicles. In this context, the DIC image or some marker to show the plasma membrane or outline of the cell is needed to show the possible intracellular localization. Maybe the intracellular localization could be studied by using some common markers of exocytosis (e.g. CD63, markers for ESCRT Pathway).

As explained in L270-273, immunofluorescence microscopy revealed the accumulation of intracellular capsids at a pericellular location (Fig 6B). The exact localization of the virus (internal or external face of the cell membrane) was not addressed in this study because it has limited biological value. It should be mentioned that the natural pH of the basolateral compartment of the airway epithelium is neutral, not acidic. Accordingly, the pericellular accumulation of viruses due to the "artificially" acidic basolateral pH is not expected to occur under the natural environment of the basolateral compartment of the airway.

This experiment, however, was useful to confirm that the neutral pH at the basolateral side of the epithelium facilitates the dissociation of the incoming viruses from globoside, which would otherwise remain irreversibly associated to the cellular membranes.

L271: Since we did not analyze whether the blocked capsids accumulated on the internal or external face of the plasma membrane, we have removed the word "intracellular".

4. Figures 3. and 5: Figure 3C. is not explained in the main text. Figure 3E mentioned in the text is missing (line 208). The explanation of Figure 3D is missing(lines 198-201).

L202 and L209: Fig. 3C and D were wrongly cited in the text. The error has been corrected.

The results of Fig 5E are not explained in the text (line 251).

L259: The results shown in Fig. 5E were explained in the text (L257-259); however, the figure was not cited in the text. We have corrected the error and Fig. 5E now appears in the text.

Please explain the results in Figs 5B, 5C, and 5D more carefully (Line 245).

L250-254: As suggested, the results in the corresponding figures are explained in more detail.

Increase the text size of Fig 5C's x-axis legend.

It was a defect in the pdf file, not in the original figures.

Does α-capsid correspond to VP2 antibody (main text line 242 and figure legend lines 629-630)?

L654: Yes, it is clarified now in the legend of figure 5.

5. In many figures, more than two samples are compared for statistically significant differences. Student's t-test is not optimal for such multiple comparisons. A better practice is to use ANOVA combined with a suitable post hoc test (for example Tukey's or Dunnett's, or maybe Games-Howell in case of unequal variances).

We agree with the reviewer that ANOVA is better suited when comparing more than two groups. However, in our figures, we compare two groups at a time. The confusion might be due to the connecting line covering various groups. To avoid confusion, we have changed the format of the connecting line to identify better the two groups that are compared.

Reviewer #3:

Introduction. p.4, li.75ff. Mention to the recently identification of AXL as a specific ligand for VP1u is appropriate in this context.

L82-84: As suggested, AXL is now mentioned in the introduction.

Material and Methods, p.17, li.439. UT7/Epo have different subclones used in different labs, please specify if cells used are the original clone or a derived subclone and provide appropriate source reference.

L456-457: As suggested, the source of the UT7/Epo cells is now provided.

Graphs in figures 1 (C, D, E), 2 (D, E), 3 (A-D), 4 (C, D), 5 (B, C, E), 6 (A), 8 (D, E, F) and S1-4 are all heterogenous and not consistent in Y-axis scale. Some axis are in log scale, some in linear scale, sometimes origins are labeled as 'zero' although clearly starting from non-zero values, sometimes origins start from non-zero values, while range and intervals of values changes from graph to graph.

All graphs show absolute values starting from 0, except for two types of experiments:

- Apical/basolateral ratio: values are exponential starting at 10e2, except for Fig. 4C and D, which start at 10e3. To homogenize the Y-axis scale, Fig. 4C and D have been modified to start at 10e2.
- Transcytosis inhibition by antibodies or drugs: values are percentage. The Y-axis of Fig. 7B has been modified to have the same interval as all the graphs with % values.

Importantly, when not expressed as percentage, figures are meant as absolute, while a normalization to cell number or medium sample volume would be appropriate to allow better comparison.

The β-actin gene was used for the normalization of data in qPCR and qRT-PCR experiments. Transcytosis results were normalized for the differences in volume between apical and basolateral compartments.

L538-539, L554-555, L562-563: For the sake of clarity, we have included a sentence in Materials and Methods.

Finally, the level of statistical significance should be stated (always missing). This heterogeneity goes to paper's disadvantage and should be amended. Moreover, although statistically significant, differences in values between experimental categories can have quite a different meaning if considered on a linear or logarithmic scale.

L576-577: As suggested, the level of statistical significance for all values is now indicated in the Materials and Methods section.

Results, p.6, li130-152 and related figure 1. Either in the legend (A) or in the Material section please indicate what secondary antibody is used to reveal the VP1u-anti FLAG complex.

L478-479: As suggested, the secondary antibody used is indicated in the Material section.

legend (C): "viral DNA was extracted and quantified by PCR" should be "DNA was extracted and viral DNA quantified by PCR".

Correction done.

In figure 1: comparing the neutral (pH 7.4/7.4) condition, the detected B19V DNA in graph C is not seen in the comparable conditions in graphs D and E (and also in figure 2D). Is this correct?

The conditions were different. Some experiments were performed at 4°C (Fig. 1C and 2D) and others at 37°C (Fig. 1E). The results were normalized for the number of cells, which can fluctuate between experiments. The difference observed is within the expected deviation.

Results, p.7, li-153-176 and related figure 2. Speaking of B3GalNT1 gene knock-out, data on sorting might be shown as supplementary results. However, in Figure 2, knock-out of gene expression is shown by end-point RT-PCR. More correctly, genome editing (knock-out) should be shown by PCR on genomic DNA, while assessment of gene expression silencing requires quantitative RT-PCR.

S2 Fig: Following the reviewer suggestion, FACS data and sorting strategy are shown as supplementary results.

We have used three gRNAs targeting different sequences in the B3GalNT1 gene. The gRNA sequences are proprietary protected, making primer design challenging and interpretation of the results confusing. The successful knockout of B3GalNT1 is demonstrated by two independent assays:

- B3GalNT1 mRNA transcripts were examined by quantitative RT-PCR, and an agarose gel is shown to confirm that the amplicons of the positive (wild-type cells) and loading control (GAPDH) are specific and have the expected size, and that the transfected cells show no detectable amplicons.
- Knockout of globoside was confirmed by confocal microscopy with a specific antibody, with the wild-type cells as positive control.

Results, p.8, li.194-201 and related figure 3. In the legend, please indicate the moi of added virus rather than an absolute amount.

For this experiment, we prefer to indicate the exact number of viral particles used. The reason is that we do not detect infection (progeny), but input particles in the apical compartment, and the progressive accumulation of particles in the basolateral compartment.

In the text, please correct reference to panels C and D (not E).

Correction done.

Although the absolute amount of virus recovered in the basolateral side is dependent on the neutral/acidic condition at the apical side, it is actually not clear whether this difference is also

present in the relative amount of virus in basolateral side compared to virus bound by cells. In other words, does the pH affects uptake or transcytosis efficiency?

pH affects uptake and, therefore, transcytosis. Under neutral pH, B19V does not bind to MDCK II or to hAECs. This is confirmed by qPCR and by immunofluorescence microscopy with antibodies against the viral capsid.

Finally, infectivity of relased virus as shown by NS mRNA should also be normalized to the amount of viral DNA in infected UT7/Epo cells.

Normalization to the amount of viral DNA would be confusing. The infection is performed at 37°C and the input samples correspond to 1h post-infection. At this time, incoming viruses are trafficking towards the nucleus, while a large proportion is being degraded in the endolysosomal compartment. Accordingly, during the first steps of the infection, the amount of incoming viral DNA shows large fluctuations among the different samples.

L538-539, 562-563: For clarity, we have added a comment to explain that the results are normalized to the number of cells by quantification of the b-actin gene, and another comment explaining that the cells were infected with the same amount of viral particles.

Results, p.11, li.260-264 and related figure 6B. I am not sure I could appreciate the difference between the two conditions, as claimed y authors. Please explain better…

In figure 6B, the distribution of the viral capsids appears to be influenced by the pH in the basolateral compartment, i.e., more pericellular when the basolateral medium is acidic, and more scattered when the basolateral pH is neutral. The two enlarged images emphasize the difference.

L276: As mentioned above, we have removed the word "intracellular" since we have not determined whether the capsids accumulate at the internal or external face of the cell membrane.

Results, p.12, li.303. Figure S8 is actually Figure S6

Correction done.

Results, p.13, li.325. Data from TEER should be shown at least as supplemental figure

S9 Fig: The values have been included as supplementary figure.

Discussion, p.15, li.372-374. Do authors hypothesize a role for the PLA2 activity associated to VP1u, especially for endosomal escape (see also p.9, li.231)

We have not tested the possibility that the PLA2 in the VP1u influences the transcellular transport. VP1u has other motifs that could explain the reduced transcytosis efficiency of particles in presence of antibodies against VP1.

Transcytosis of cargo occurs inside endosomal vesicles and does not involve membrane disruption. Accordingly, we do not think that during transcytosis the virus employs the PLA2 activity to escape from endosomes, as it occurs during the infection to target the nucleus. However, since we have not experimentally addressed the intracellular pathway followed during the transcellular transport, we prefer to avoid speculation on this point.

Discussion, p.15, li.380-382. The mentioned intracellular conditions required for productive infection are effective in the nucleus of infected (erythroid progenitor) cells, while in the present experiments it seems that virus does not enter nucleus of epithelial cells, and the observation does not seem relevant.

We agree with the reviewer. However, we cannot exclude that a minor proportion of intracellular viruses targets the nucleus. That is why we explain that, even in the case that some incoming viruses would reach the nucleus, the lack of EPO signaling would make replication unlikely.

L349-351: For clarity, we have modified the sentence, and added that the receptors involved in the entry and trafficking of B19V in the airway epithelium are distinct from those employed to infect the erythroid progenitor cells.

Discussion, p.16, li. 400-402. Can authors comment on this statement comapared to what shown in Bonsch et al, J.Virol 2008, 82:11784-91.

The blood sample used in that study (Bonsch et al, J.Virol 2008, 82:11784-91) was acidic due to anaerobic glycolysis, a process that occurs naturally during long blood storage. Under low pH conditions, most of the circulating viruses bind to globoside expressed in RBCs. This has been explained in our previous study (Bieri et al., PLoS Pathog. 2021 17(4):e1009434) and cited in this study.

Discussion, p.16, li.404-407. The statement is only partly justified by the present experiments, and in any case absence of globoside would block not only transcytosis but also as amplification and presentation to the immune system following a productive infection.

L423: To follow the reviewer's suggestion, we have modified the sentence and use the word "support" instead of "explain".

Part III – Minor Issues: Editorial and Data Presentation Modifications
Please use this section for editorial suggestions as well as relatively minor modifications of existing data that would enhance clarity.

Reviewer #1:

NA

Reviewer #2:

1. Figs the statistical significance P values should be explained either in the Figure legends or in the materials and methods e.g. For all P values, significance levels are denoted as follows: *P < 0.05, **P < 0.01, and ***P < 0.001.

L576-577: As suggested, the P values appear in the Materials and Methods section.

2. The Fig 1A. Please, add an explanation of what is shown in the images.

As suggested, more information is provided in the legend of Fig. 1A

3. Line 137. Please explain when first mentioned what term "a VP1u cognate receptor means".

To avoid confusion, "cognate" has been changed for "erythroid"

4. Line 159, 161: Explain the globoside knockout MDCK = KO MDCK when first mentioned.

Done

In the figure legend 2A "and transfected cells (KO)" should be "globoside KO transfected cells"

Done

Lines 167-170 sounds like materials and methods.

Sentence has been moved to the legend of Fig. 2

5. Line 170: While B19V DNA was …

Done

6. Figure legend S2A. Could you please explain what is ZO since it is used first time here.

ZO-1, zonula occludens. It was already explained in a previous figure (Fig. 4; L648).

7. Line 205. It might be good to explain that when treated at 85 °C the capsids were most likely disintegrated and their genome was exposed to nuclease.

L744: As suggested, a comment was added.

8. Line 215: Explain TC - tricellular contact?

TC: Tissue culture. It is explained in L182

9. Line 253-254, 264-265, 295-296: add space

We do not find a problem in those lines.

10. Fig 7B. Add the units of the used chemical concentrations to the x-axis legends since they are not explained elsewhere.

Units have been added.

How can transcytosis of the virus be more than 100% in some of the treatments? If this is due to the experimental set-up, please explain it more carefully in the results section.

This is because the untreated sample was set to 100%. The values above 100% are within the standard deviation.

L683-684: An explanation was added to the figure legend.

11. Line 303: Should Figure S8 be S6?

Yes, correction done.

12. Line 310: What does the cell body mean? What about just cells? Line 358 etc.:

The cilia belongs to the cell. “Cell body” is used to distinguish between the ciliated part and the rest of the cell.

13. The figures should not be mentioned in the Discussion.

As suggested, citations to figures are removed from the Discussion section.

14. Supplementary figure legends: Please mention the cells used.

L727: We have added the cell type used.

Reviewer #3:

(No Response)
