# Supplementary material for: Globoside and the mucosal pH mediate parvovirus B19 entry through the epithelial barrier
Source: PLoS Pathog. 2023 May 23;19(5):e1011402. doi: 10.1371/journal.ppat.1011402 (PMC10241365; doi:10.1371/journal.ppat.1011402)
Supplement: Attachment — Submitted filename: Response to reviewers_Suter et al.docx [file ppat.1011402.s011.docx]

Reviewer's Responses to Questions

**Part I - Summary**
Please use this section to discuss strengths/weaknesses of study, novelty/significance, general execution and scholarship.

Reviewer #1: The authors appear to have addressed all of the raised concerns, and as a result, the manuscript now appears suitable for publication in PLoS Pathogens.

Reviewer #2: The authors have responded to all the reviewer comments and revised the manuscript accordingly.

Reviewer #3: I confirm my overall appreciation of the submitted manuscript, the paper is of high value, and a source for future research in the field. On general terms, considering the actual revision, my opinion is that authors could have been more compliant to reviewers’ suggestions. I acknowledge that almost all of my own suggestions have been replied in the appropriate way, nonetheless still I would ask for amendments in a few instances, critically concerning graphs.

**Part II – Major Issues: Key Experiments Required for Acceptance**
Please use this section to detail the key new experiments or modifications of existing experiments that should be absolutely required to validate study conclusions.

Generally, there should be no more than 3 such required experiments or major modifications for a "Major Revision" recommendation. If more than 3 experiments are necessary to validate the study conclusions, then you are encouraged to recommend "Reject".

Reviewer #1: (No Response)

Reviewer #2: No additional experiments are required.

Reviewer #3: None

**Part III – Minor Issues: Editorial and Data Presentation Modifications**
Please use this section for editorial suggestions as well as relatively minor modifications of existing data that would enhance clarity.

Reviewer #1: (No Response)

Reviewer #2: No additional comments.

Reviewer #3: Concerning data presentation in graphs, although normalization is now mentioned by authors in the methods section and figure captions, most figures still report quantities on Y-axis as absolute numbers. Except for % values, these still need to be expressed as normalized to some other reference quantity (e.g., number of cells, viral genome copies, etc…).

Absolute values were obtained with an standard curve. Standard curves are built using serial dilutions of a known amount of plasmid molecules containing the target sequence. The unknown samples are compared with the standard curve to obtain absolute values.

**Line 531-532**. We have included a comment to clarifiy this point. “Absolute quantification was performed using the standard curve method”.
